# Gender Differences in Perception of Romance in Chinese College Students

**DOI:** 10.1371/journal.pone.0076294

**Published:** 2013-10-16

**Authors:** Jie Yin, John X. Zhang, Jing Xie, Zhiling Zou, Xiting Huang

**Affiliations:** 1 Key Laboratory of Cognition and Personality (Ministry of Education), Southwest University, Chongqing, China; 2 Department of Psychology, Fudan University, Shanghai, China; Wake Forest School of Medicine, United States of America

## Abstract

Women often complain that their partners are not romantic enough. This raises the question: how romance is recognized and evaluated in a love relationship? However, there has been essentially no empirical research bearing on this issue. The present set of studies examined possible gender differences in perceptions of romance and the associated neural mechanisms in Chinese college students. In Study 1, 303 participants (198 women, 105 men) were administrated a questionnaire consisting of 60 sentences and required to rate the romance level of each sentence. Results showed higher rating scores in males than females for low romance items, but not for high or medium romance items. In Study 2, 69 participants (37 women, 32 men) were recruited to judge the degree of romance in sentences presented on a computer screen one by one. Compared with females, males again showed higher scores and responded more slowly only to low romance items. In Study 3, 36 participants (18 women, 18 men) currently in love with someone were scanned with functional MRI while they did the romance judgment task from Study 2. Compared with females, greater brain activation was found for males in the frontal lobe, precentral gyrus, precuneus and parahippocampal gyrus for low romance items. The results provide the first piece of evidence for gender differences in romance perception, suggesting enhanced cognitive processing in males when evaluating the degree of romance in romantic scenes.

## Introduction

Love is generally regarded as one of the deepest and most meaningful sentiments. The study of love has a long history, starting several decades ago. For example, Rubin had done research on the measurement of love versus liking [1]. Among the various types of love, romantic love is said to have inspired some of the greatest achievements of mankind and is considered a culturally universal and powerful experience that affects many aspects of human life [2], [3]. Romantic love is highly correlated with relationship satisfaction, relationship quality and stability [4], [5], and is often a prerequisite for marriage [6]. Directed towards a single person, it is a complex sentiment involving cognitive, emotional, behavioral and erotic components that may vary on the basis of gender [7], [8].

Subsequently, Hatfield and Walster [9] distinguished between romantic love and companionate love in emotion intensity, sexual arousal and relationship stability. In Sternberg’s triangular theory of love [7], romantic love involves more intimacy and passion, but less commitment than other types of love. Hendrik and Hendrik [10] speculated that people go through a developmental sequence of love styles, with romantic love (Eros) developed around early adulthood. Others suggest that romantic love is influenced by attachment styles formed in childhood [11], [12].

With the development of functional brain imaging technology, researchers have begun to explore the neural mechanisms related to romantic love. Activation in reward and motivation systems has been observed when participants gaze at photographs of their lovers [13]–[15]. Evidence showed that activations observed in romantic love were similar to that for maternal love [16], possibly because they share a common and crucial evolutionary purpose, the maintenance and perpetuation of the species. Rejection in romantic love has also been studied. Subjects showed brain activity changes in cerebellum, anterior temporal cortex, insula, anterior cingulate, and prefrontal cortex during acute grief when they alternated between recalling a sad, ruminative thought about their loved one and a neutral thought about a different person they know for an equally long time [17]. When participants viewed images of lovers who had rejected them, there were increased activations in cortical and subcortical regions such as orbitofrontal cortex, insular cortex and anterior ventral pallidum associated with reward evaluation, craving and addiction, emotion-related learning and behavior control [18]. These studies on the neural mechanisms underlying romantic love focused mainly on the emotional components and have not reported any gender differences [14]. This was perhaps due to the small number of participants and unequal gender distribution in these studies, for example, 11 females and 6 males in Bartels’s study [14].

Previous studies on behavioral and erotic components of romantic love have demonstrated some gender differences. Generally, in love relationships, males pursue their female partners more actively, while females are more passive [19], [20]. Men tend to fall in love more “easily” than women [21]. Compared with females, males mentioned romance more frequently [22]. The number of male university students visiting internet pornography sites is twice that of female students [23]. Several studies have shown that many college-age women engage in unwanted sexual activity with a dating partner [24]–[26]. These findings demonstrate that in love and sexual relationships males generally take the initiative.

Regarding the cognitive and rational components of romantic love, there has been some preliminary evidence for gender differences. For example, in evaluating romantic attraction, men describe themselves as being more attracted to physical appearance, while women show higher level of attention to intimacy, commitment and security [27]–[29]. The two genders also have different preferences for partners’ personalities. Males prefer women who are virtuous, understanding, obedient and who have good ability to nurse, while females prefer men who are diligent and ambitious [30]. Other research has found that males and females had different attitudes toward love and sexuality. For example, women had more negative implicit attitudes toward sexuality than did men [31]. Male respondents held more permissive attitudes toward premarital sex than did females, and both males and females expressed greater permissiveness to male premarital sexual behaviors [32].

Chinese have long been regarded as non-romantic [33]. Gao found that passion was significantly higher in US couples than in Chinese couples [34]. Research has also shown that Japanese are less romantic than Russians or Americans [35]. Cultural psychologists indicate that collectivistic (Eastern) and individualistic (Western) cultures have significant differences in how romantic love is experienced and valued [36], [37]. In the Chinese culture, romantic relationships involve long-term commitment and the requisite seriousness for commitment, but affection between couples is not intensive [38]. In comparison, Americans experience stronger emotion in a relationship [37], [39].

In modern Chinese culture, an interesting daily phenomenon is that women often complain that their partners are not romantic enough [40], even though males usually take the initiative in relationships. We suspect that this problem may be related to an ability we call “romance perception”, defined here as an individual’ ability to recognize romantic situations, analogous to the visual perception of, for example, color and shape. Just as a man with relatively better visual perception would identify objects more easily, a man with better romance perception would be more likely to sense romance in a particular setting. Someone weak in romance perception may not perceive romance in scenes that others consider romantic. More specifically, romance perception may include the ability to identify romance in various real life situations. It may also depend on the criteria that an individual adopts in judging how romantic an event is.

From the observation that females often complain of lack of romance in their partners, it is possible that women may have lower romance perception and do not feel as romantic as men do in the same situations. To test this possibility, we first constructed a romance scale consisting of sentence items describing romantic scenes and asked college students who were in love to evaluate the romance level of such items. We then conducted a behavioral experiment to test college students’ romance perception. We expected to find that females would evaluate the same items as less romantic than males. Due to lack of literature on this topic, our expectation was based more on a speculation than any *a priori* hypothesis.

In case that males and females do differ in romance perception, it would be necessary to identify the underlying mechanisms. There are gender differences in cognitive and emotional processes as women tend to perform at a higher level than men do on most verbal tests while men outperform women on visual-spatial tasks [41]. In cognitive control of emotion, females show more activation in regions associated with emotional processes and males show more activation in regions related to cognitive processes [42]. In addition, men and women show activation differences in emotion regulation [43]. Harenski et al. found that when viewing unpleasant pictures and rating their degree of moral violations, females showed stronger activity in the posterior cingulate and insula, and males showed increased activity in inferior parietal regions. Females tended to adopt care-based evaluations while males tended to adopt justice-based moral evaluations when making moral judgments [44].

This literature suggests that males may judge the romantic quality of X (scenes/situations/scenarios…) based on rational analysis, while females base their judgments more on the emotions evoked. Given that the romance perception tasks we would use involve both emotional and cognitive processing, we expected that males would show stronger activation in cognition-related brain regions when evaluating the degree of romance in various scenarios, while females would show stronger activation in emotion-related regions.

## Methods

### Ethics Statement

Out of all participants in this research, only one was a minor. We obtained her parents’ verbal informed consent via telephone because they lived in a province far away from the university. The Institutional Review Board at Southwest University (SWU) in Chongqing, China approved this consent procedure. Written informed consent was obtained from all other participants. The Institutional Review Board at SWU approved all procedures.

### Study 1

#### Participants

Three hundred and three undergraduates (198 females, 105 males; age range from 17 to 24 years; mean age = 20.5years) were recruited from SWU mainly via flyers seeking students who were currently in love with someone. Other participants were recruited by word of mouth in classes. The gender groups were matched in age, handedness, and education (female: age 20.4±1.1 yrs; male: age 20.9±0.9 yrs). Among them, 164 participants were in love with someone, and the remaining 139 were not. Each received a small gift for compensation for their time after the study.

#### Materials and procedure

Based on literature review and pilot studies, we first constructed a set of items that possibly described a romantic event, including scenes and behaviors. With further check-up and fine tuning, the items were compiled to make a romance perception questionnaire using a 10-point Likert self-report scale. There were a total of 60 items, each describing an event that may frequently occur when a romantic relationship is forming, such as “I recall the scene we kissed each other for the first time”. Higher scores indicate a higher degree of romance for the item (0 = not romantic at all, 9 = highly romantic).

The scale was then given to the 139 participants who were not in love with someone. They were instructed to evaluate the degree of romance of every item. Based on their rating, the 60 items were sorted in ascending order and equally divided into 3 levels of high (e.g., “We back-to-back sit on the flat roof with the number of stars”), medium (e.g., “I call him/her every day”), and low romance rating (e.g., “He/she asks what gift I want for my birthday”). The rate of agreement in classifying items into levels of romance was 95% between “in love” and “not in love” subgroups. Therefore, there was not a significant difference in the romance scale determined from the “not in love” subset and the entire participant pool. There was one minor reason we prefer using only input from the “not in love” participants. With the romance scale determined from people in a neutral state (i.e., not in love) and then used for people in a love state, independence between scale development and scale application was maintained.

ANOVA analysis showed that the rating scores were different across categories (F_(2,276)_ = 567, p<0.001). Post-hoc comparisons indicated that the mean rating score of high romantic items (M = 6.73, SD = 1.3) was significantly higher than medium romantic items (M = 5.67, SD = 1.32) (p<0.001), and the mean rating score of medium romantic was significantly higher than low romantic items (M = 3.77, SD = 1.03) (p<0.001).

The Cronbach’s Alpha was 0.94 for the whole questionnaire, and 0.92, 0.89, and 0.77 for the high, medium, and low levels respectively. Inter-correlation between levels was significant: 0.84 between high and medium levels, 0.60 between medium and low levels, and 0.41 between high and low levels.

#### Results

All 164 participants returned the questionnaires but 32 were discarded due to missing data, leaving 132 valid data (55 males, 77 females). To balance gender distributions, we randomly selected 54 males and 57 females from the data, equating mean age and education. The following results were based on these 111 participants.

For romance perception, a 2 (gender: male, female)×3 (romance level: high, medium, low) analysis of variance (ANOVA) revealed a significant interaction between gender and romance level (F_(2, 218)_ = 11.47, p<0.001). The main effect of romance level was significant (F_(2,218)_ = 529.12, p<0.001), but the main effect of gender was not (F<1). In a separate analysis where relationship length was entered as a covariate, the results were similar.

**Figure 1:**
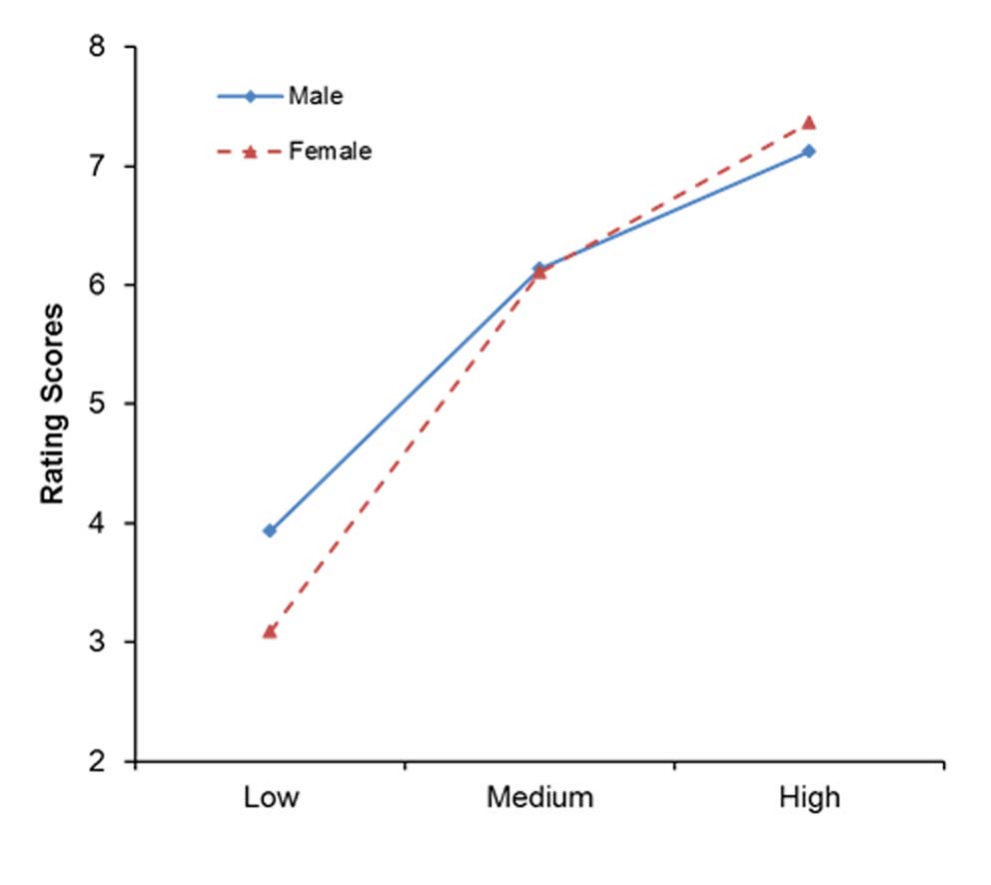
Ratings of romance degree across three stimulus levels in males and females. Males rated significantly higher than females for Low romantic stimuli.

As shown in Figure 1, post-hoc comparisons indicated that there was a gender difference at the low romantic level (t = 3.63, p<0.001), i.e., the mean score was higher for males (M = 3.93, SD = 1.21) than for females (M = 3.09, SD = 1.23). That is, males showed higher romance perception than females for low romantic events but the two genders did not differ for high or medium romantic events.

#### Discussion

The results were in accord with our expectation showing higher romance perception in males than females for low romantic events but no gender differences for high and medium romantic events. This pattern indicated that some events considered romantic by the males may not be considered so by the females. That is, females may have higher threshold in the evaluation of romantic events. This may account, to some extent, for their complaints that their partners are not romantic enough.

This finding is consistent with research showing that males are more romantic than females [45], [46]. Men perceive romance with a lower threshold, so they may think some events frequently happening in a love relationship as very romantic. However, women may not feel as much romance in some scenes, for example, “We go out for dinner going Dutch”.

### Study 2

In this study, we intended to verify the finding in Study 1 with a behavioral test involving not only romance perception but also response time measures. We intended to see if there were gender differences in reaction times (RTs) when evaluating the romantic stimuli.

#### Participants

Thirty-seven female and 32 male undergraduates (female: age 21.65±1.40 yrs; male: age 21.97±1.98 yrs) were recruited from SWU by flyers and internet advertisement. All were in love with someone, and the length of love relationship ranged from 1 to 38 months (mean length: 13.07±9.15 months). The two gender groups were matched in age, handedness, education and love state (score of Passionate Love Scale). We also examined some of the confounding factors, including age, relationship length, residence (rural or urban), social class, and distance (long distance or not), but did not identify any significant gender differences. Each participant signed a written informed consent and was paid 20 RMB for compensation of their time.

#### Materials and procedure

**Figure 2:**
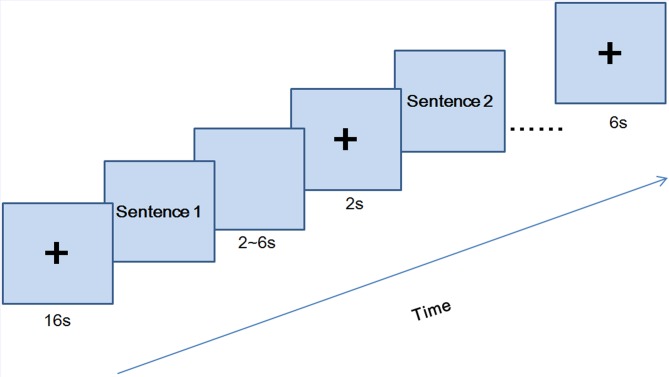
The time course of experimental paradigm in Study 2.

A “Romance Perception Task” (RPT) was administered to all participants assessing the ability to perceive and evaluate romantic scenes. The same 60 sentences in Study 1 were presented using E-Prime software (Psychology Software Tools, Inc. Pittsburgh, PA, USA) in two runs, each lasting approximately 10 min. Two runs using all 60 sentences were completed and they differed only in sentence presentation order. As shown in Figure 2, in each trial, a sentence was presented until there was a response, followed by a blank screen of variable duration (2, 4, or 6 s) and then a fixation (2 s). Participants were instructed to press buttons in the keyboard indicating “How romantic do you think the situation is?” (from 0 to 9, 0 = not at all romantic, 9 = extremely romantic). Each sentence was presented only once in each run.

#### Results

Two females and one male were excluded due to extremely long RTs (>5000 ms). We performed repeated-measures ANOVA with sentence type as a within-subject factor.

**Figure 3:**
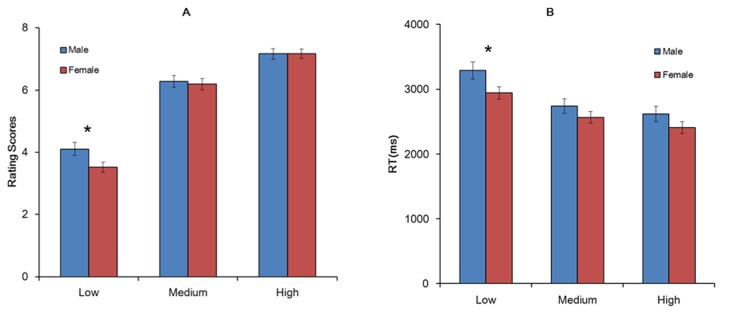
Rating scores (A) and reaction times (B) over types of stimuli (low, medium, and high romance levels). Significance bars and asterisks designate the significance of rating and reaction time for the gender by stimulus type interactions. *, p<0.05.

As shown in Figure 3A, there was a significant main effect of stimulus type (F_(2,128)_ = 637.51, p<0.001) and gender by stimulus type interaction (F_(2,128)_ = 5.28, p<0.01) on ratings of romance. Males rated low romance sentences higher than females did (p<0.05) but no gender differences were observed for medium or high romance sentences (Fs<1). The RT data showed similar results (Figure 3B). Males responded significantly more slowly to low romantic stimuli than females did (p<0.05), but there were no gender differences for the medium or high romantic stimuli (gender × stimulus-type interaction, F _(2, 128)_ = 2.95, p<0.05). A separate analysis including relationship length as a covariate showed similar results.

#### Discussion

Rating results for the present study replicated findings from Study 1 showing gender differences when rating low but not medium or high romance sentences. The RT results showed a similar pattern, suggesting that males may need more effort to evaluate the degree of romance in low romance situations. Females, possibly being more familiar with these romantic events, rated them low and spent less time in decision.

### Study 3

This study was conducted to understand more about the gender differences observed in the above two studies by examining the neural mechanisms involved in romance perception.

#### Participants

A new group of 18 female and 20 male undergraduates from the SWU participated in the study, recruited through advertisement in university BBS. Two males were excluded due to large head motion (>3 mm), leaving 18 females and 18 males for final analysis. The two gender groups were matched in age, handedness, and education. All were in an intensive love relationship scoring high on the Passionate Love Scale, (PLS) (i.e., higher than 4.8 on a 6-point Likert scale; M = 5.20, SD = 0.27) [47]. For this sample, the length of love relationship ranged from 4 to 48 months (M = 18.42, SD = 10.65). All participants were right-handed and none reported any neurological or psychiatric disorders. Pre-screening interviews were conducted to verify that they were heterosexual (self-reported as having only opposite-sex sexual desire and partners). Each signed a written informed consent and was paid 50 RMB for their time.

#### Stimuli and procedure

The 60 sentence items in the questionnaire from Study 1 were used, each presented in black color on a white background. Participants pressed buttons to indicate their evaluation of the degree of romance of each sentence. E-prime software was used for visual stimulus presentation and experimental control. Responses were recorded using a fiber-optic finger-switch response system.

**Figure 4:**
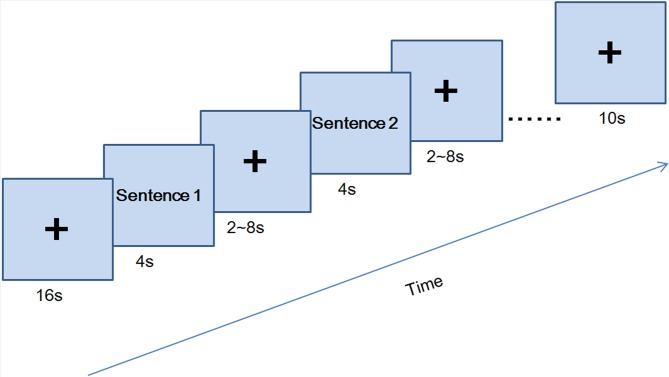
The time course of experimental paradigm in Study 3.

Each participant completed 2 runs. In each run, all 60 items (20 for each type: high, medium, low romance levels) were presented in pseudorandom order. Run 2 was a repeat of run 1 (with different stimulus presentation order) to obtain more data point. For each run, as shown in Figure 4, there was a 16 s fixation prior to the first item and a 10 s fixation after the last one. Each item was presented for 4 s, followed by a variable fixation (2, 4, 6, or 8 s). Using variable duration is a common practice in event-related fMRI designs to enhance the power of the regression analysis and to reduce participants’ expectation of incoming stimuli. The three types of items (high, medium, low romantic level) were in pseudorandom order.

Before scanning, each participant was asked about the start time, duration, intensity, and feelings of their love relationship. They also completed the PLS to test their intensity of passion for the relationship. Participants were instructed to evaluate degree of romance for each item via a button press (from 1 to 4) (1 to 4; 1 = not at all romantic, 2 = slightly romantic, 3 = very romantic, 4 = extremely romantic) when viewing the sentences on the screen. Four keys were used as opposed to the 10 keys in Study 2 due to physical constraints inside the scanner.

#### Post-scan interview

After the scanning session, we conducted exit interviews to determine whether participants followed the instructions and assess how they felt during the fMRI scan. All reported performing reasonably well with little head movement.

#### Image Acquisition and Data Analysis

Data were acquired using a 3-T Marconi (Siemens) Edge MRI system. Blood-oxygen -level-dependent (BOLD) responses and in-plane anatomical data were recorded for each participant. Images were (a) anatomical, axial T1-weighted spin-echo scans: 2.52 ms TE, 1900 ms TR, 9° ﬂip angle, 25 cm FOV, 1 mm slice thickness, 256×256 matrix size, 32 slices; (b) functional, T2* Gradient-Echo EPI scans: 30 ms TE, 2000 ms TR, 90° ﬂip angle, 22 cm FOV, 3 mm slice thickness, 64×64 matrix size, 32 slices. Voxel size for functional images: 3.4 × 3.4 ×3.0 mm.

Brain Voyager QX software (version 2.0) was used for fMRI data analyses. The functional data were first pre-processed, via procedures consisting of slice scan time correction, 3D motion correction (We excluded participants whose head motion was more than 3 mm), and temporal data smoothing with a high-pass filter of three cycles in the time course and spatial smoothing. Each participant’s high-resolution anatomical image was normalized into standardized Talairach coordinate space [48]. The normalization process in Brain Voyager consists of two steps: an initial rigid body translation into the AC-PC plane, followed by an elastic deformation into the standard space. The resulting set of transformation was applied to the preprocessed functional image, followed by spatial smoothing (Gaussian kernel, full-width at half-maximum of 6.0 mm).

Each stimulus sentence (high, medium, low romance perception) was treated as a separate regressor and modeled as a boxcar function convolved with the canonical hemodynamic response function. We applied a high-pass filter with a cut-off of 2 sines to remove low-frequency signal components. Motion covariates were removed. Contrast images were created for each comparison for each participant and then combined across-participants in mixed-effects general linear models, treating participant as a random effect and gender, stimulus type as fixed effects.

Using whole-brain analysis, pairwise comparisons were conducted between different romance level at a threshold of p<0.05 (FDR corrected) and a cluster size of 50 voxels. For gender differences, brain activations were compared between males and females at a threshold of p<0.05 (FDR corrected) and a cluster size of 20 voxels.

#### Results

##### Behavioral results

For reaction times, a 2×3 (gender by romance level) ANOVA revealed a main effect for romance level (F_(2,68)_ = 89.05, p<0.001). All pairwise comparisons were significant with response to the low romance items (M = 2377 ms, SD = 304) vs. medium romance items (M = 2098 ms, SD = 281), and medium romance items vs. high romance items (M = 1939 ms, SD = 289). There was no significant interaction between gender and romance level (F<1). The main effect of gender was not significant (F<1). For all three romance levels, RT was shorter for females than for males, though the difference was not significant.

For rating scores, there was a marginal interaction effect between gender and romance level (F_(2,68)_ = 2.72, p = 0.07), a main effect of romance level (F_(2,68)_ = 368.04, p<0.001). The main effect of gender was not significant (F_(1,34)_ = 1.88, p = 0.18). In addition, the ANOVA analysis using relationship length as a covariate showed similar results.

##### Imaging results

ANOVA results identified significant main effects for romance level, gender, and their interaction in the right precuneus, right superior frontal gyrus and left cerebellum (p<0.05). As shown in Table 1, high romance items led to greater activation than medium romance items in cingulate gyrus and precuneus. The opposite contrast led to activation in left superior frontal gyrus and right precentral gyrus. High romantic items led to greater activation than low romance items in the bilateral posterior cingulate gyrus, left postcentral gyrus, left parahippocampal gyrus, and right anterior cingulate. The low minus high romance contrast activated the frontal gyrus, temporal gyrus, right thalamus and right insula, in addition to the same set of regions in the medium minus high contrast. The low minus medium comparison revealed more activation in the frontal gyrus, the left middle temporal gyrus and the left cerebellum. The medium minus low comparison revealed stronger activation in left postcentral gyrus.

**Table 1:** Areas of activation sensitive to romance levels.

			BA	x	y	z	Voxels	*t*max
**High>Medium**								
right anterior cingulate			33	3	17	16	1972	5.104
left precuneus			7	−3	−40	43	3074	6.310
**Medium>High**								
right precentral gyrus			4	39	−19	52	4679	6.838
left superior frontal gyrus			6	−3	8	52	1693	6.650
**High>Low**								
right cerebellum,culmen				27	−43	−17	4634	6.368
right posterior cingulate			29	6	−46	7	1448	5.086
right anterior cingulate				0	41	1	3464	6.343
left cingulate			31	−9	−31	34	6246	7.580
left posterior cingulate			29	−9	−49	10	1984	6.756
left postcentral gyrus			3	−33	−22	46	15157	8.709
left parahippocampal gyrus				−30	−34	−8	1354	6.441
left superior occipital			19	−36	−76	28	1651	4.503
left postcentral gyrus			40	−63	−25	22	1373	5.570
**Low>High**								
right insula			22	45	−28	1	1588	5.501
right precentral gyrus			4	33	−25	52	34955	13.270
right inferior frontal			47	42	29	−2	13073	7.140
right thalamus				12	−13	10	8617	8.781
left cerebellum, declive				−9	−73	−8	56791	9.942
left superior frontal gyrus			6	−3	11	52	35758	8.918
left inferior frontal gyrus			45	−48	23	7	31820	10.840
left superior temporal gyrus			39	−54	−58	22	3326	5.965
**Medium>Low**								
left postcentral gyrus			3	−33	−22	43	7578	7.316
**Low>Medium**								
right insula				30	20	1	17511	7.647
right supramarginal gyrus			40	51	−52	28	4734	7.113
right precentral gyrus			4	33	−22	58	22626	8.207
right medial frontal			9	3	44	22	23698	8.002
right thalamus				9	−7	13	5137	7.550
left cerebellum, declive				−12	−73	−11	36240	7.601
left thalamus				−9	−10	13	1944	5.279
left middle temporal gyrus			21	−57	−4	−5	16940	6.563
left inferior parietal			40	−45	−49	28	4199	6.089

*x,y,z*, Talairach coordinates (Talairach and Tournoux, 1988) of the peak voxel of the activated cluster. *t* max, *t* value of the maximally activated voxel within the cluster.

#### Gender differences

**Figure 5:**
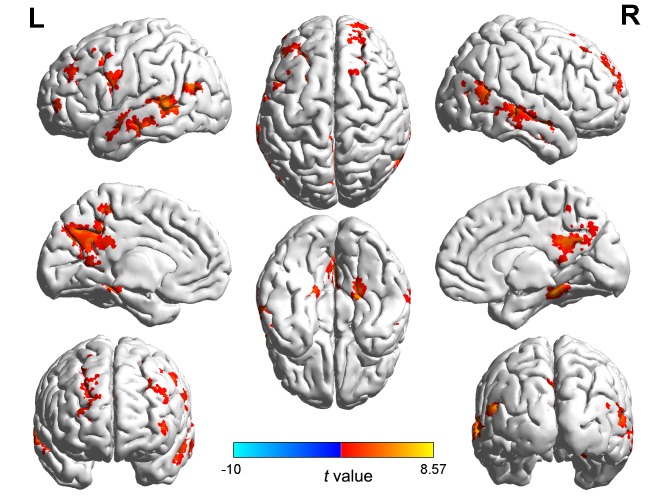
Areas of activation affected by sex differences (Male>Female) pooled over all romance levels.

**Figure 6:**
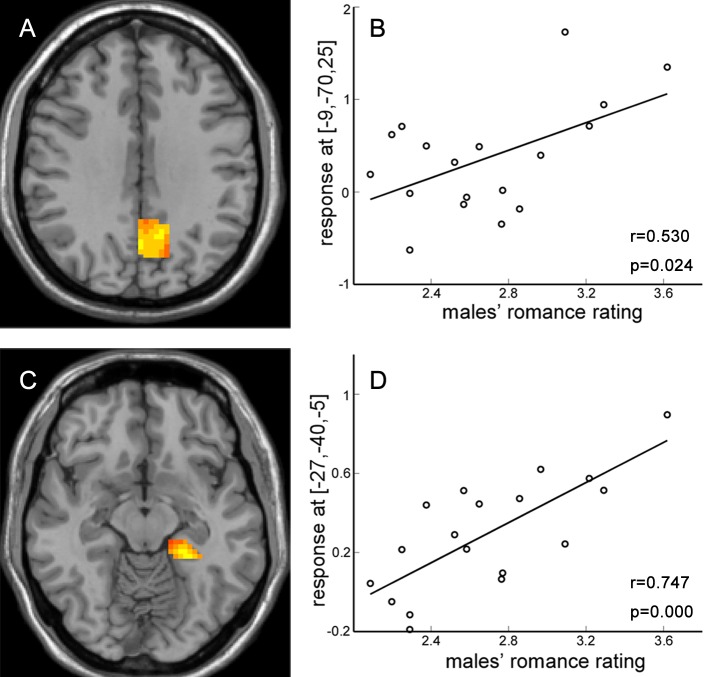
Males’ romance rating scores correlated with activation in specific regions. A: the left precuneus location for the correlation. B: correlation of activity in the left precuneus with males’ rating scores. C: left parahippocampal gyrus location for the correlation. D: correlation of activity in the left parahippocampal gyrus with males’ rating scores.

In relation to gender differences (Table 2 and Figure 5), males showed greater activation than females in the frontal lobe (especially middle and superior frontal gyrus), precentral gyrus, precuneus, fusiform gyrus, posterior cingulate cortex and parahippocampal gyrus. We also examined the correlation between brain activation and romance rating scores and identified two regions showing significant positive correlations for males: the left precuneus (−9, −70,25) and left parahippocampal gyrus (−27, −40, −5) (See Fig. 6). In a partial correlation analysis controlling for relationship length, the brain regions correlated with males’ romance rating scores were still the left precuneus (p = 0.026) and left parahippocampal gyrus (p = 0.001).

**Table 2:** Areas of activation sensitive to sex differences (male>female).

			BA	x	y	z	Voxels	*t*max
right superior frontal gyrus			8	30	41	38	758	4.827
right middle temporal gyrus			39	36	−67	22	646	5.237
right fusiform gyrus			37	36	−43	−14	1554	5.510
right posterior cingulate			23	3	−37	25	1031	5.065
left precuneus			31	−9	−70	25	6160	6.186
left parahippocampal gyrus			36	−27	−40	−5	958	6.112
left middle temporal gyrus			19,21	−36	−79	19	1452	4.989
left middle frontal gyrus			46	−52	35	19	771	5.416
left precentral gyrus			6	−60	2	13	596	6.359

*x,y,z*, Talairach coordinates (Talairach and Tournoux, 1988) of the peak voxel of the activated cluster. *t* max, *t* value of the maximally activated voxel within the cluster.

#### Gender differences in Low romantic level

**Figure 7:**
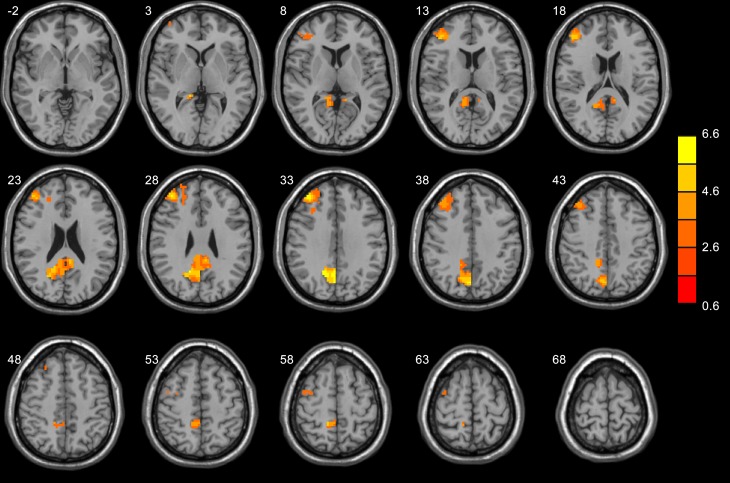
Areas of activation for low romance level (Male>Female).

Simple effect analysis indicated males had stronger activation in more regions than females for low romance sentences. These brain regions were localized in the right middle frontal gyrus, right precuneus, bilateral posterior cingulate gyrus, as shown in Table3 and Figure 7. The males vs. females contrast did not reveal activation in any region for medium or high romance items using the same threshold (FDR corrected, p<0.05, 10 voxels cluster size). That is, gender differences were found only for the low romance level. All the ANOVA analysis using relationship length as a covariate showed similar results.

**Table 3:** Areas of activation sensitive to sex differences at low romance level (male>female).

			BA	x	y	z	Voxels	*t*max
right middle frontal gyrus			8	27	35	40	475	4.017
right precuneus			7	3	−73	34	2674	5.840
right posterior cingulate			23	3	−37	25	340	4.265
left posterior cingulate			30	−12	−52	10	449	4.489

*x,y,z*, Talairach coordinates (Talairach and Tournoux, 1988) of the peak voxel of the activated cluster. *t* max, *t* value of the maximally activated voxel within the cluster.

#### Discussion

The behavioral results in the fMRI study showed the same trend as in Study 2 but not reaching significance, possibly due to the small sample size.

The high minus low romance comparison showed activations in regions associated with emotion, including cingulate gyrus, parahippocampal gyrus, and left postcentral gyrus [49]. For example, cingulate cortex activation was found in viewing photographs of one’s lover [14], [50]. These results indicate that high romantic stimuli elicited stronger emotional response than did low romantic stimuli. In addition to emotion-related regions, the left postcentral was also activated, possibly reflecting responses to printed words [51], [52].

The low minus high romance comparison showed a large set of activations, including frontal gyrus, temporal gyrus, right precentral gyrus, thalamus and cerebellum. Coupled with effects for RT, this suggests that evaluations of romance were more difficult or requiring extensive processing in social cognition for low romance items than high romance items. The temporal regions are known to be important in perceiving socially relevant information such as gaze and expression [53]–[55]. Similarly, the frontal lobe is critical in linking perceptual representations with representations of their emotional and social significance [56]. The high minus medium romance comparison resulted mainly, in increased activation of cingulate gyrus, a structure implicated in emotion. The result was consistent with the small behavioral differences between the two conditions. As with the low minus high and the low minus medium romance comparisons, there were similar activations related to social cognition, such as frontal gyrus, temporal gyrus, supramarginal gyrus and thalamus [57].

Considering effects of gender, activation differences revealed from the male minus female comparison were typically related to cognitive processing, including regions in frontal lobe, temporal gyrus, precuneus, fusiform, parahippocampal gyrus and posterior cingulate cortex. The precuneus is a key part of the neural substrate for visual imagery [58]–[60]. It shows consistent activation for recollection judgments of previously studied words in an fMRI episodic retrieval study [61]. Its activation may reflect reinstatement of visual images associated with remembered words. Several recent studies provide new insights into how the parahippocampal cortex (PHC) represents space [62], [63]. Mullally and Maguire’ studies showed the PHC is responsive to the awareness of surrounding local space suggesting a fundamental role in scene processing. In our study, male participants may have reinstated the actual scenes related to them and their lovers when viewing the sentences about romantic events. However, females may not engage such processing. Given the known connection between fusiform gyrus and face processing [64]–[67], the male participants may recall the face of their lovers during romance evaluation. In addition, these romantic items activated more emotion-related regions in males than females.

The results from simple effect analysis showed that large gender differences appeared at low romance level only. These regions included frontal cortex, precuneus and posterior cingulate cortex. Clearly, the imaging results were consistent with the behavioral results in Study 1 and 2 showing no significant gender differences at high and medium romance levels.

We expected males would have stronger activation in cognition-related regions and females would show heightened activity in more emotion-related regions during romance evaluation. The results supported this expectation partially. From the brain activation pattern, more regions for cognitive processes (and some related to emotional arousal) were engaged for males than for females when evaluating romance events. This was also confirmed in the correlation analysis where BOLD responses in two regions, left parahippocampal gyrus and left precuneus showed significant correlation with males’ romance rating scores.

### General discussion

In this set of studies, we used several methods including questionnaire, behavioral testing, and brain imaging to explore how male and female Chinese college students differ in romance perception and the underlying neural substrates for the observed gender differences. As far as we know, this is the first empirical study on romance perception.

Study 1 and 2 show that males tended to rate an event as more romantic and took longer times to make romance judgments than did females in the low romantic items. This suggests that females have higher criteria in romance judgment. There is evidence that in daily life, females pay more attention to the emotional aspects of an event, and are more interested in romantic stories in literature and movies [68]–[72]. That is, females are more familiar with romantic events and also have higher expectation of romance. In comparison, males are not as sensitive or pay less attention to emotional stimuli, including romantic scenes, and have less experience in romance judgments. Compared with females, males’ judgments of low romantic items as more romantic indicate their low criteria in romance perception. On the other hand, with relatively weaker ability to perceive romance, males would be less skillful in differentiating different levels of romance, resulting in their slower reaction time in romance level judgment. That is, it may be more cognitively effortful for males to evaluate romance levels of events in light of their lower overall focus on romance.

Study 3 indicates that exposure to sentences depicting low romantic situations activated more regions involved in social cognition, while exposure to sentences describing high romantic events activated more emotion-related areas. These results suggest that high romantic sentences may be so prototypical of romance ideals that they are recognized and evaluated automatically, arousing emotional responses but not engaging deliberate cognitive processing. In comparison, sentences of low romantic events may require more deliberate and cognitive processing during evaluation. This was also shown from the much slower responses to the low romantic items than to the high romantic items.

These results also demonstrate that as other social cognition tasks, both emotional and cognitive processing was involved in the present romance perception task. Somewhat consistent with our results, in close relationship situations, females are known to exhibit stronger activation in emotion-related areas, and tend to adopt care-based evaluation, whereas males show stronger activation in areas associated with cognition and tend to adopt justice-based evaluation [42], [44]. Consistent with this general finding in the literature, our imaging results showed that more cognitive brain regions were activated in males than in females. In addition, in males, BOLD responses correlated significantly with romance rating scores in two regions, precuneus and parahippocampal gyrus. This result provides more support to a previous point that romance evaluation may be more effortful to males, as suggested by their longer RTs. Furthermore, the fact that males showed greater activations in emotional regions such as the posterior cingulate suggests that the present task of romance evaluation led to more emotion arousal in males compared with females.

However, we did not find any emotion-related areas showing greater activation in females than in males or any significant correlations between activity in emotional regions and females’ romance rating scores. This may be due to stimulus format. Apparently, verbal descriptions of romantic events may not be the most effective way in eliciting extensive emotional processing, compared to, for example, the presentation of pictures or video clips. We speculate that the mode of stimulus presentation would affect males less whose imaging and RT results suggest that romance judgments were based more on cognitive processes.

The gender differences observed here may not generalize beyond Chinese samples given the high dependence of social cognition on culture. Individualist and collectivist cultures differ in many respects, including norms guiding intimate relationship [34], [36]. Research has also found cultural differences in affect and cognition [73]–[76]. It remains to be seen whether and how males and females differ in romance perception in other cultures.

As in studies of hetero-sexual couples, a potential confounding factor in the present study is the actor/partner effect. That is, it is unclear whether male (or female) participants showed different response patterns because they were male (or female) or because they had a female (or male) partner. Whether the gender effect found here reflects an actor or partner effect (or both) can only be sorted out with future research, perhaps with homosexuals or with designs explicitly manipulating heterosexual interactions.

The present study is also limited by other potentially cofounding variables. For example, relationship length for Study 3 ranged from 4 to 48 months. People in different relationship stages differ in mental processes and neural responses to romance. Future work may reveal concomitant variations in romance perception by focusing on more limited relationship durations of participants. Other variables such as whether the relationship was long-distance and previous experience in love relationships may also affect results and warrant consideration in future studies. Furthermore, the present conclusion may be specific to the age range of our participants. Whether it also applies to older population such as people between 40–60 years of age would be an interesting issue to be addressed in future research.

For romance level, there was no particular reason to have 3 levels as opposed to 2 or 4 levels although we think using three categories was neither too coarse nor too fine in differentiating the items. The results in Study 1 were similar when dividing into four or two levels. For four levels, the gender difference was significant in the lowest level (p<0.001), but not the other upper levels. For two levels, the gender difference was marginally significant in the lower level (p = 0.07), but not significant in the upper level. Other than convenience and power consideration, dividing the items into three levels was in a sense arbitrary, although this arbitrariness was acceptable for an explorative study identifying new effects to be scrutinized by future research.

## Conclusions

Briefly, the present results show that when evaluating romance, male Chinese college students tended to give higher rating than females for low romantic events. High romantic events engaged brain regions implied in emotional processing but low romantic events engaged brain regions associated with cognitive processing. Low romantic events were rated as more romantic and activated more brain regions in males than in females. The results showed enhanced cognitive processing in males, possibly due to their lack of experience in processing romantic information. In comparison, appraisals of romance in females may be more automatic, possibly relying on emotional processing.

## Supporting Information

Appendix S1 (DOC)
