# Supplementary material for: Gender Differences in Perception of Romance in Chinese College Students
Source: PLoS One. 2013 Oct 16;8(10):e76294. doi: 10.1371/journal.pone.0076294 (PMC3797815; doi:10.1371/journal.pone.0076294)
Supplement: Appendix S1 — (DOC) [file pone.0076294.s001.doc]

Appendix S1: Examples of stimuli

High romance degree group: I have a gorgeous candlelit dinner with him/her; We go for a stroll hand in hand; We back-to-back sit on the flat roof with the number of stars.

Medium romance degree group:  I call him/her every day; We do the cleaning together; We always have a date in the cafe.

Low romance degree group：I kiss him/her on a public occasion; We have a dinner going Dutch; He/ she asks what gift I want for my birthday.
